# Integrative immunology identified interferome signatures in uveitis and systemic disease-associated uveitis

**DOI:** 10.3389/fimmu.2025.1509805

**Published:** 2025-04-09

**Authors:** Danielle Dias Munhoz, Dennyson Leandro M. Fonseca, Igor Salerno Filgueiras, Haroldo Dutra Dias, Helder I. Nakaya, Igor Jurisica, Hans D. Ochs, Lena F. Schimke, Luiz Vicente Rizzo, Otavio Cabral-Marques

**Affiliations:** ^1^ Experimental Biology Laboratory Prof. Dr. Geraldo Medeiros-Neto, Hospital Israelita Albert Einsten, Sao Paulo, Brazil; ^2^ Interunit Postgraduate Program on Bioinformatics, Institute of Mathematics and Statistics (IME), University of Sao Paulo (USP), Sao Paulo, SP, Brazil; ^3^ Department of Immunology, Institute of Biomedical Sciences, University of Sao Paulo, Sao Paulo, Brazil; ^4^ Department of Neuroscience, Institute of Biomedical Sciences, Federal University of Minas Gerais (UFMG), Belo Horizonte, Brazil; ^5^ Division of Orthopaedics, Osteoarthritis Research Program, Schroeder Arthritis Institute, and Data Science Discovery Centre for Chronic Diseases, Krembil Research Institute, University Health Network, Toronto, ON, Canada; ^6^ Departments of Medical Biophysics and Computer Science, and Faculty of Dentistry, University of Toronto, Toronto, ON, Canada; ^7^ Institute of Neuroimmunology, Slovak Academy of Sciences, Bratislava, Slovakia; ^8^ Department of Pediatrics, University of Washington School of Medicine, Seattle, WA, United States; ^9^ Seattle Children’s Research Institute, Seattle, WA, United States; ^10^ Network of Immunity in Infection, Malignancy, and Autoimmunity (NIIMA), Universal Scientific Education and Research Network, (USERN), Sao Paulo, Brazil; ^11^ Department of Clinical and Toxicological Analyses, School of Pharmaceutical Sciences, University of Sao Paulo (USP), Sao Paulo, Brazil; ^12^ D’Or Institute for Research and Education (IDOR), São Paulo, Brazil; ^13^ Department of Medicine, Division of Molecular Medicine, Laboratory of Medical Investigation 29, University of São Paulo, Sao Paulo, Brazil

**Keywords:** non-infectious uveitis, blood transcriptome, interferon-regulated genes, cytokines, integrative analysis

## Abstract

**Introduction:**

Uveitis accounts for up to 25% of global legal blindness and involves intraocular inflammation, classifed as infectious or non-infectious. Its complex pathophysiology includes dysregulated cytokines, particularly interferons (IFNs). However, the global signature of type I, II, and III interferon-regulated genes (Interferome) remains largely uncharacterized in uveitis.

**Methods:**

In this study, we conducted an integrative systems biology analysis of blood transcriptome data from 169 non-infectious uveitis patients (56 isolated uveitis, 113 systemic disease-associated uveitis) and 82 healthy controls.

**Results:**

Modular co-expression analysis identified distinct cytokine signaling networks, emphasizing interleukin and interferon pathways. A meta-analysis revealed 110 differentially expressed genes (metaDEGs) in isolated uveitis and 91 in systemic disease-associated uveitis, predominantly linked to immune responses. The Interferome database confirmed a predominance of type I and II IFN signatures in both groups. Pathway enrichment analysis highlighted inflammatory responses, including cytokine production (IL-8, IL1-β, IFN-γ, β, and α) and toll-like receptor signaling (TLR4, TLR7, TLR8, CD180). Principal component analysis emphasized the IFN signature’s discriminative power, particularly in systemic disease-associated uveitis. Machine learning identified IFN-associated genes as robust predictors, while linear discriminant analysis pinpointed CCR2, CD180, GAPT, and PTGS2 as key risk factors in isolated uveitis and CA1, SIAH2, and PGS in systemic disease-associated uveitis.

**Conclusion:**

These findings highlight IFN-driven imune dysregulation and potential molecular targets for precision therapies in uveitis.

## 1 Introduction

Inflammatory intraocular disorders encompass a wide range of conditions in which the eye or its components are targeted by the immune system, leading to significant visual impairment (1). One prominent example is uveitis, a chronic, organ-specific disease characterized by inflammation of the uvea, the eye layer between the sclera and retina. Irreversible lesions mark this condition and carry a significant risk for progressing to blindness (2, 3). Uveitis may also involve inflammation affecting adjacent intraocular structures such as the retina, vitreous, and optic nerve (4–6). It is estimated that over two million individuals worldwide are affected by uveitis, with its incidence varying significantly depending on the region, potentially accounting for 10-15% of all cases of preventable blindness (7–9).

Uveitis can be categorized based on its etiology into infectious and non-infectious types (10, 11), although the pathogenic mechanisms of the latter are not yet fully understood. Non-infectious uveitis, devoid of any identifiable infectious agent, can emerge within the context of ocular or systemic syndromes, such as Behçet disease, sarcoidosis, psoriatic arthritis, juvenile idiopathic arthritis, Vogt-Koyanagi-Harada (VKH) disease, and ankylosing spondylitis (AS), or it may manifest solely within the eye as the primary site (4–6, 12, 13). The latter manifestation often displays features of an autoimmune or autoinflammatory disease (14). The aberrant activation of innate immune processes, triggered by endogenous danger signals, metabolic mediators, or cytokines, can lead to local tissue damage even in the absence of a specific antigenic target, resulting in uncontrolled and dysregulated host immune responses (15).

While non-infectious uveitis comprises diverse manifestations, understanding the etiology and heterogeneity of uveitis phenotypes remains limited. Previous reports addressing the pathogenesis of uveitis have implicated T-helper type 1 (Th1) and Th17 lymphocytes and their predominantly produced cytokines, interferon-gamma (IFN-γ) and interleukin (IL)-17, respectively, as the cause of exacerbated inflammation associated with uveitis (3, 16).

Recent evidence suggests that the different forms of uveitis arising from autoinflammatory or autoimmune processes may share a common molecular and immunopathogenic mechanism affecting IFN signaling (17–20). It is noteworthy that although IFNs can induce the pathologic process, they can also be suppressed in non-infectious uveitis, as some uveitis patients had low serum levels of type I IFN (21–23), suggesting alternative disease mechanisms among the different forms of uveitis.

Interferons are cytokines secreted by distinct immune cells and are pivotal mediators of innate and adaptive immune responses. They also play a crucial role in the onset and progression of immune-mediated diseases, including inflammatory and autoimmune disorders (24, 25). The global signature of type I, II, and III interferon-regulated genes, called interferome, represents a well-established facet of the immune responses across various contexts (26, 27). A comprehensive interferome database has been developed to compile interferon-regulated genes (IRGs) identified in previously published *in vitro* experiments in which multiple cell types were treated with different types of interferons, including IFN type I (α, β, δ, ϵ, ζ, κ, ν, τ, ω), type II (IFN-γ), and type III (IFN-λ) (26). This database enables the exploration of IRG signatures through cross-referencing with gene lists derived from transcriptomic or proteomic analyses.

Despite its recognized role in immune regulation, a comprehensive analysis of the interferome signature in uveitis remains lacking. To advance our understanding of uveitis pathophysiology, we conducted an integrative analysis of multiple blood transcriptome studies from uveitis patients, accounting for disease heterogeneity to characterize its distinct interferome profile.

## 2 Materials and methods

### 2.1 Data curation

We systematically searched the Gene Expression Omnibus genomics data repository (GEO; https://www.ncbi.nlm.nih.gov/geo/) to collect publicly available gene expression data. The applied search query was *uveitis[MeSH Terms] OR uveitis[All Fields] OR autoimmune uveitis[MeSH Terms] OR autoimmune uveitis[All Fields]) AND Homo sapiens[Organism] AND Expression profiling by high throughput sequencing[DataSet Type] OR Expression profiling by array[DataSet Type].* This search identified 12 studies published between November 2009 and January 2023. RNAseq and MicroArray studies were included in the integrative analysis.

The inclusion criteria comprised: (1) *Homo sapiens* microarray or RNAseq expression data, (2) non-infectious uveitis datasets, (3) whole blood, peripheral blood mononuclear cells (PBMC), or specific cellular subtype samples (**Supplementary Table S1**). The exclusion criteria included (1) infectious uveitis, (2) nonhuman samples, and (3) *in vitro* or stimulated cells. Six datasets were included in the analysis: GSE66936 (28), GSE194060 (29), GSE195501 (29), GSE166663 (30), GSE18781 (31) and PRJNA702017 (32).

169 blood transcriptomic samples from uveitis patients and 82 healthy control (HC) samples were analyzed. Samples were categorized into two groups according to disease characteristics: 1) Uveitis only (non-infectious and no additional reported syndromes), 2) Systemic disease-associated uveitis (uveitis as a symptom of systemic diseases such as Behçet disease, sarcoidosis, VKH disease, AS, Inflammatory Bowel Disease (IBD), tubulointerstitial nephritis). Information about the included series is provided in **Supplementary Table S1**.

### 2.2 Analysis of gene co-expression modules

Gene co-expression analysis was performed employing the R package CEMiTool and using default parameters (33) in R studio Version 4.3.2 2023 (RStudio; https://www.rstudio.com). This allowed us to identify groups of genes that exhibit correlated expression patterns across samples from each of the datasets of the uveitis only group and the systemic disease-associated uveitis group, respectively. Representative results from one dataset of each group are displayed, and enriched gene sets and pathways for selected modules are demonstrated by interactive gene networks and bar plots using default graphical packages.

### 2.3 Transcriptional meta-analysis and gene annotation

A comprehensive meta‐analysis of gene expression datasets was conducted using NetworkAnalyst 3.0, employing Fisher’s p-value combination method (34). The meta-analysis was performed separately for the two groups of uveitis (uveitis only: GSE66936, GSE194060, GSE195501, and systemic disease-associated uveitis: GSE166663, GSE18781, PRJNA702017). Using empirical Bayes regression (through ComBat) the data sets of both groups were adjusted for batch effect and visualized by principal component analysis (PCA) and on Density plots. The gene expression distribution of meta-significant genes (metaDEGs) based on the average Log_2_ Fold Change (FC) is depicted across each biological process for the top 10 enriched biological pathways, visualized by Ridgeline charts.

### 2.4 Interferome analysis

The interferome database V2.01 (http://www.interferome.org/interferome/home.jspx) was utilized to analyze the IFN network. This database compiles IRGs identified in prior experimental studies involving human and nonhuman samples. The metaDEGs of each group obtained in our previous analysis were cross-referenced with the interferome database, and only IRGs associated with previous experimental studies on human samples were chosen for subsequent analysis (**Supplementary Tables S2**, **S3**). Intersections of Type I, II, and III IRGs for each respective uveitis group were selected using Venn diagrams created with the Bioinformatics & Evolutionary Genomics online tool (https://bioinformatics.psb.ugent.be/webtools/Venn/).

### 2.5 Functional enrichment

Functional enrichment analysis was performed for each set of intersecting IRGs from both uveitis groups using the ClusterProfiler (35) R package version 4.3.1. The enriched Gene Ontology (GO) Biological Processes (BPs) were filtered based on an adjusted *p*-value < 0.05 (Benjamini-Hochberg test), and the most significant BPs were visualized using a bubble heatmap created with the ComplexHeatmap R package (36, 37). Additionally, Pathway Association Prediction (PAP) analyses were conducted through Pathway Data Integration Portal (pathDIP) (38), version 5.0.33.1 (https://ophid.utoronto.ca/pathDIP) to further explore the pathway associations of the intersecting IRGs. Detailed results are available in **Supplementary Tables S4**–**S6**.

### 2.6 Molecular network

A comprehensive network and interactome analyses were performed using the Integrated Interactions Database (39) and NAViGaTOR (40) software to visualize physical protein-protein interactions between the identified IRGs and key components of the JAK-STAT signaling pathway. The list of IRGs from each uveitis group was separately utilized as input for the Integrated Interactions Database (IID ver. 2021-05; https://ophid.utoronto.ca/iid). The resulting network was annotated and visualized using the NAViGaTOR ver. 3.0.19. The final network figure was generated from the SVG output file using Adobe Illustrator ver. 28.5.

### 2.7 Principal component analysis

Principal Component Analysis (PCA) was performed to evaluate the capacity of the interferome to differentiate between uveitis patients and healthy controls within each respective uveitis group, using the previously identified IRGs. Eigenvalues and eigenvectors exceeding one intercept (41) were essential to demonstrate group segregation. PCA by singular value decomposition (42, 43) was conducted using the scaled expression values of the IRGs (**Supplementary Tables S2**, **S3**) utilizing R packages factoextra (44), ggplot2 (45), and ggExtra (46).

### 2.8 Random forest modelling

The Random Forest (RF) model was employed to rank the most pertinent genes for effectively classifying uveitis patients within each disease group using the R package randomForest version 4.7-1.1 (47). This machine learning algorithm utilized five thousand classifier trees (48) to discern predictive genes based on their scaled expression levels. The mean minimum depth, Gini decrease, and the number of appearances in nodes were utilized as criteria for determining variable importance in the classification. For cross-validation, we allocated 75% of the data for training and 25% for testing. Model quality was assessed through the out-of-bags (OOB) error rate and the Receiver Operating Characteristic (ROC) curve analysis. The expression profile of the top 15 genes, as ranked by RF, were visualized using a heatmap generated by the R package ComplexHeatmap (37) and Circlize (49), according to the gender and age of individuals within the healthy control group and the two uveitis groups. Boxplots were created using the R package ggplot (45) to compare gene expression levels across the defined groups, disease status, and gender. Statistical significance was assessed using the Wilcoxon test, with *p*-values adjusted for multiple comparisons. Linear regression was applied to evaluate the relationship between age and gene expression levels, with separate control and patient group analyses. Scatter plots with regression lines and Pearson correlation coefficients were generated using R packages ggplot (45) and ggpubr (44).

### 2.9 Linear discriminant analysis

Linear Discriminant Analysis (LDA) was conducted to determine a specific gene’s Odds Ratio (OR) in classifying an individual into one of the uveitis groups based on scaled gene expression levels. This method involves identifying a linear combination of variables (genes) capable to characterize two or more classes of objects/events (50), i.e., healthy controls vs. uveitis patients within either the uveitis only group or systemic disease-associated uveitis group. To differentiate the healthy control group from the disease groups we identified genes with a specificity and sensitivity value of approximately 70% as threshold. Based on this threshold, the detection values of each gene were categorized from 0 to 1 in each group. The analysis used the R package MASS (51) with the *lda* function. The specificity and sensitivity of the group prediction were visualized using the R package pROC (52). Plots generated from this analysis were created using the R package meta (53).

## 3 Results

### 3.1 Modular co-expression analysis of blood transcriptomes in uveitis patients identifies key cytokine signaling networks

**Figure 1:**
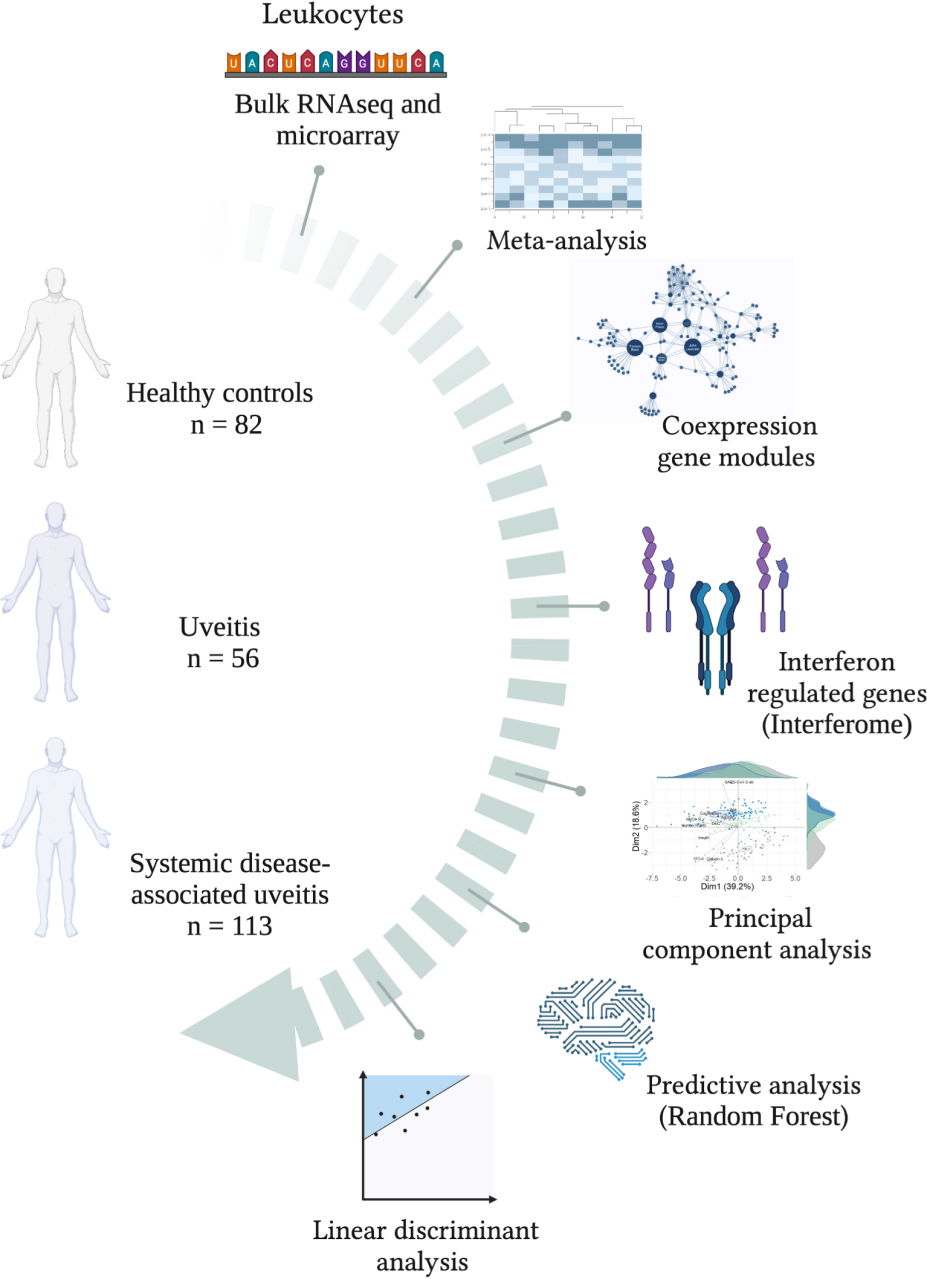
Study workflow. A schematic overview of groups and bioinformatics analyses for characterizing the interferome signature in patients with uveitis, subdivided into groups of uveitis only and systemic-diseases­ associated uveitis. Created with BioRender.com.

A comprehensive multi-study analysis (**Figure 1**) of a non-infectious uveitis cohort was performed to characterize the gene expression signature in the blood of patients with uveitis either as a sole clinical manifestation group (uveitis only) or associated with systemic diseases. Therefore, six datasets of blood transcriptomes from uveitis patients were included and divided into two separate groups: (1) uveitis only, with patients suffering from non-infectious uveitis without other systemic conditions; (2) systemic disease-associated uveitis group, with patients presenting with varied systemic disease manifestations, uveitis being one of them.

**Figure 2:**
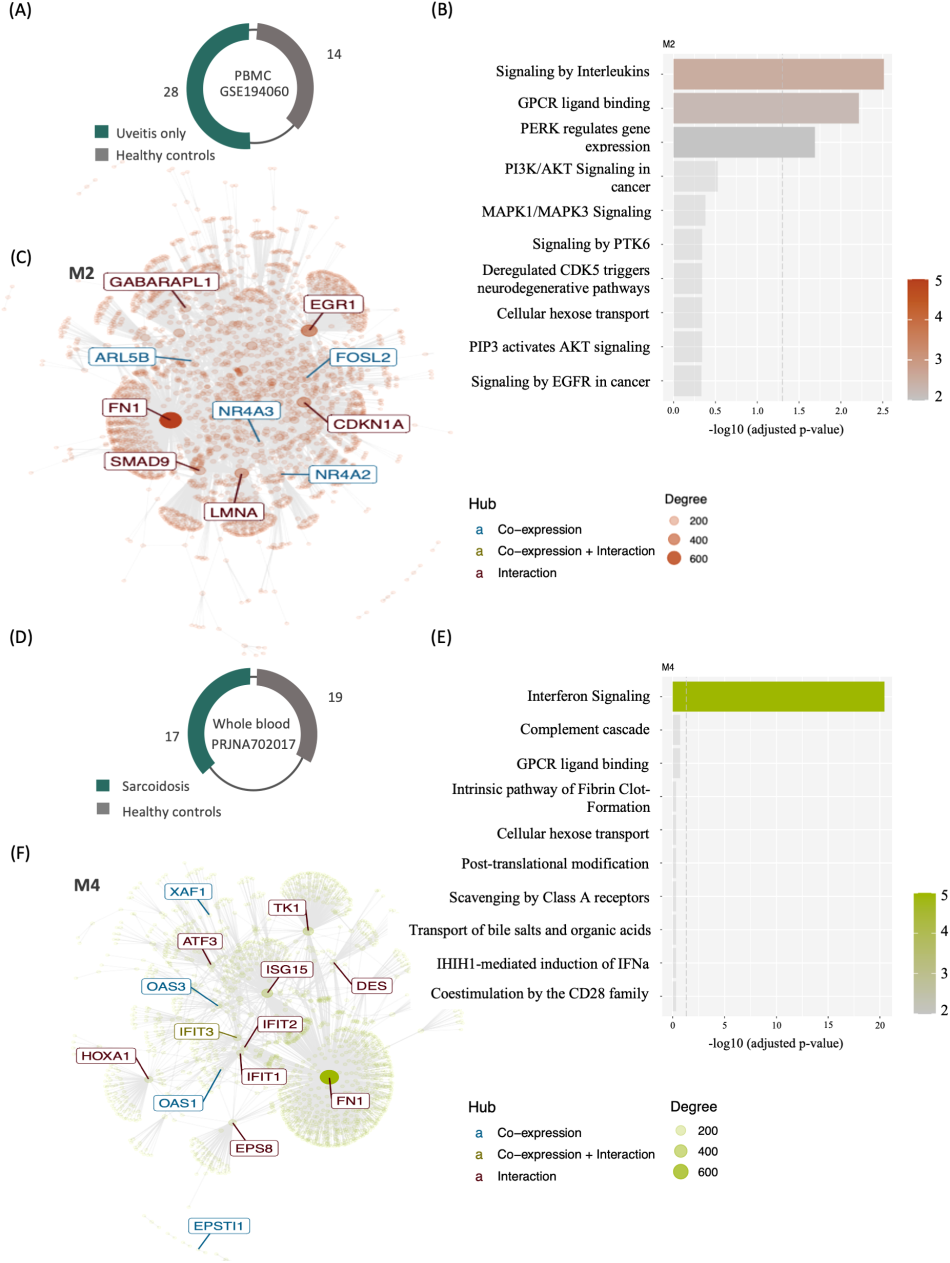
Modular gene co-expression networks in uveitis patients and healthy controls’ blood transcriptomes. **(A)** Schematic representation indicating the sample sizes for the uveitis only group (dataset GSE194060) and **(D)** systemic disease-associated uveitis (Sarcoidosis subgroup of dataset PRJNA702017). **(C)** Interaction plot for M2 (GSE194060) and **(F)** for M4 (PRJNA702017), which includes genes enriching various cytokine signaling pathways. The most interconnected genes (hubs) are highlighted within rectangles. The node size corresponds to its degree of interactivity. The bar plot illustrates the top 10 enriched pathways from the over-representation analysis of module M2 **(B)** and M4 **(E)**.

To gain insights into the systemic function of genes expressed in leukocytes, we carried out a modular co‐expression evaluation by performing enrichment and network analyses of the uveitis only and the systemic disease-associated uveitis group. A representative dataset from each group is displayed in **Figure 2**. Both datasets revealed several co-expressed genes that either interact with each other or are similarly co‐regulated during the immune response of uveitis. Among them, cytokine-associated modules were identified when comparing uveitis patients to healthy control individuals, specifically interleukin signaling on module M2 (**Figures 2A, B**) and interferon signaling on module M4 (**Figures 2D, E**). These modules’ most interconnected genes (hubs) are highlighted in an interactive network (**Figures 2C, F**). The data point toward a systemic involvement of immune response components and a strong association between interleukins and interferon signaling during the immunopathological process of uveitis.

### 3.2 The expression of IFN-associated genes predominates in the leukocyte transcriptome of uveitis patients

**Figure 3:**
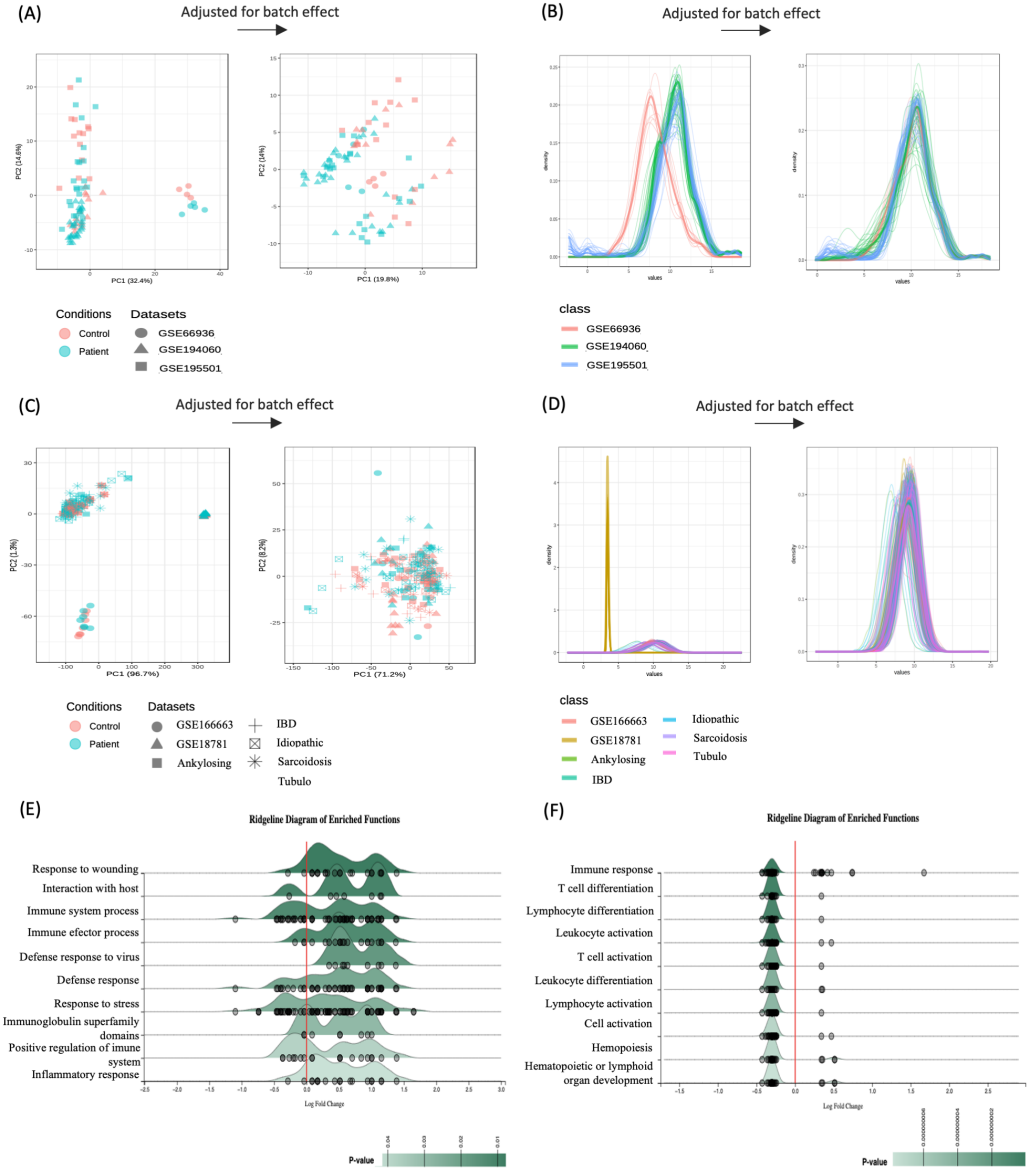
Integrative meta-analysis. Batch effect adjustment via empirical bayes regression (using ComBat) visualized by principal component analysis (PCA) for **(A)** uveitis group and **(C)** systemic disease-associated uveitis group, with density plots for **(B)** uveitis only group and **(D)** systemic disease-associated uveitis group. Ridgeline chart illustrating the Fold-Change (FC) distribution of the top 10 enriched pathways by meta-significant genes of **(E)** uveitis group and **(F)** systemic disease-associated uveitis group. Gene expression distribution (represented by small gray circles) is based on the average Log2 FC across enriched biological processes.

A meta-analysis incorporating all datasets of each uveitis group, while adjusting for batch effects, was performed to pinpoint differentially expressed genes (DEGs) across these distinct uveitis forms (**Figures 3A–D**). This analysis resulted in 110 metaDEGs for the uveitis only group and 91 metaDEGs for the systemic disease-related uveitis group (**Supplementary Tables S7**, **S8**). For instance, metaDEGs of the uveitis only group comprise genes that are involved in the innate immune response, such as chemokine receptors (CX3CR1, CCR1, and CCR2), and Toll-like receptors (TLR4, TLR7, TLR8, and the TLR4 homologous receptor, CD180). In addition, genes such as *P2R2Y*, *PELI1*, and *PTGS2* (COX-2) encoding proteins that play an essential role in inflammatory processes were present as metaDEGs (**Supplementary Table S7**). In the systemic disease-associated uveitis group, the identified metaDEGs play pivotal roles in immune responses, notably implicating T and B cell activation alongside cytokine signaling pathways. Of particular significance are genes such as *TRBC2*, *TRDC*, *LCK*, *IL1R2*, *IL2RB*, *IL7R*, and *GBP5* that are associated with the regulation and orchestration of immune reactions, such as antigen recognition by T cells and other activation processes, and the regulation of cytokines production such as interferons and interleukins (**Supplementary Table S8**). The enrichment analysis of these respective metaDEGs unraveled mostly immune system processes among the top 10 enriched BPs, including activation of defense responses, inflammatory processes, and lymphocyte, especially T cell activation (**Figures 3E, F**).

Besides the involvement of chemokines, TLRs, and T and B cell activation, we identified a direct association with the interferon signaling pathway in both uveitis groups. Several metaDEGs from the uveitis only group, including IFIT3, IFI44, IFITM1, MX1, TLR7 and DHRS9 (**Supplementary Table S7**), and gene hubs from the modular co-expression analyses of the systemic disease-associated uveitis group (**Figure 2F**) are critical players in interferon-induced immune responses. These data point toward a similar systemic immunopathological mechanism regulating IFN signaling in patients with uveitis.

### 3.3 IFN type I, II, and III signatures in uveitis immunopathology

**Figure 4:**
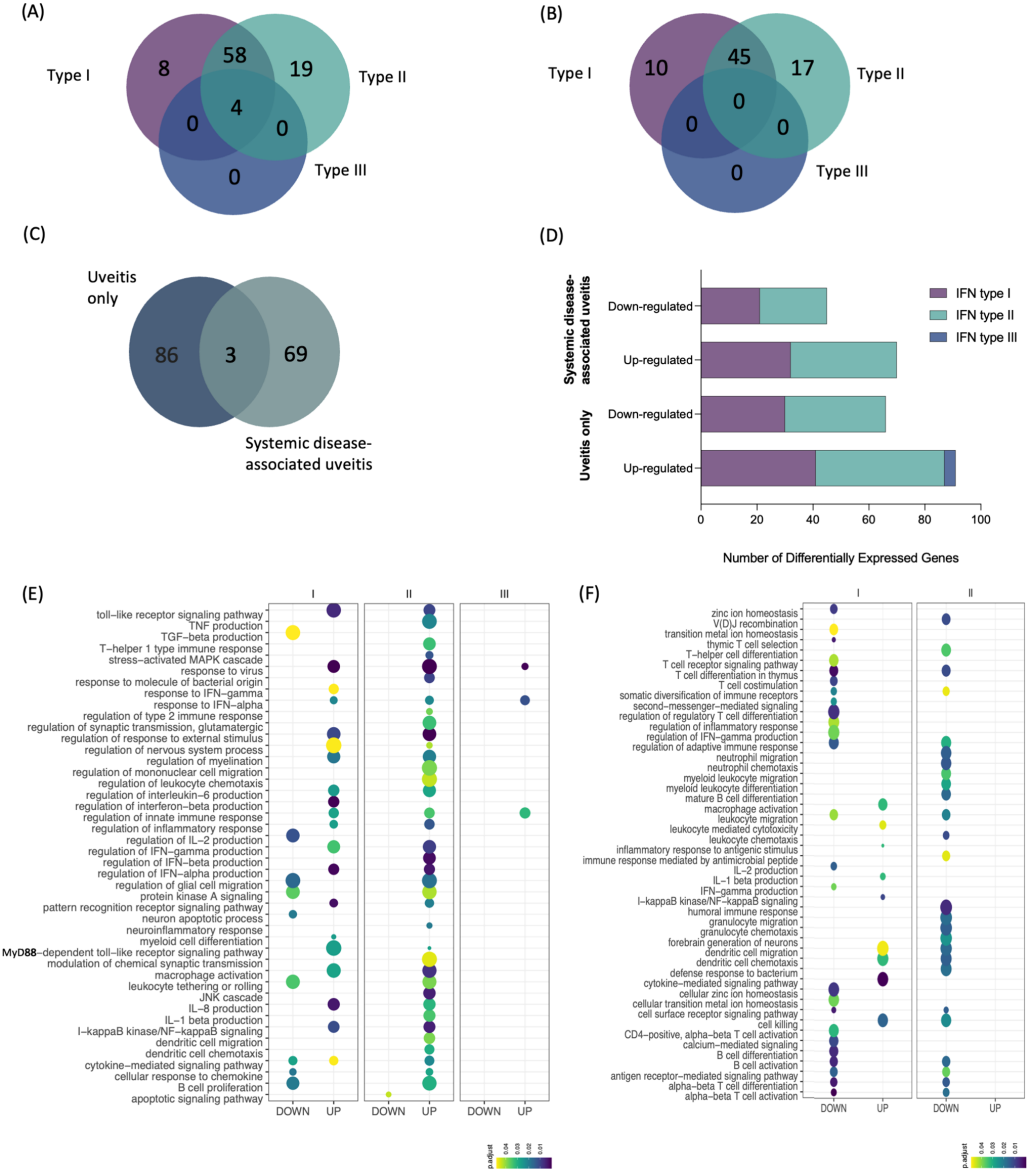
Intersection and enrichment analysis by interferon types. **(A)** Venn diagram showing the intersection of type I, II, and III interferon-associated genes [list of genes in **Supplementary Material**: (**Supplementary Tables S2**, **S3**)] of **(A)** uveitis group and **(B)** systemic disease-associated uveitis group. **(C)** Venn diagram showing the intersection of IRGs between both uveitis groups. **(D)** Total number of differentially expressed genes by type of IFN and disease group. Bubble heatmap representing the top-ranked combined scores for biological processes related to interferon-associated genes of **(E)** uveitis only group and **(F)** systemic disease-associated uveitis group. The circles' size and color correspond to – log10-transformed adjusted p-value and combined score, respectively. Rows and columns were clustered based on Euclidean distance between combined score values.

Based on these findings, we assessed the extent of the interferon signature in the immunopathology of both uveitis groups using the Interferome database, an open-access resource containing genes regulated by types I, II, and III interferons (27). This approach revealed that among the 110 metaDEGs identified in the uveitis-only group, 89 genes were classified as IRGs. Most of these genes (n = 85) were regulated by IFN type I and/or II, eight genes being regulated exclusively by IFN type I and 19 genes exclusively by IFN type II. In contrast, only four genes were concomitantly regulated by IFN types I, II, and III (**Figure 4A**). For the systemic disease-associated uveitis group, 72 genes out of 92 metaDEGs were identified as IRGs, all regulated by IFN types I and/or II, with ten genes exclusively regulated by IFN type I and 17 genes exclusively regulated by IFN type II. None were regulated by IFN type III (**Figure 4B**). Importantly, only three IRGs (CR1, CST7, and GNLY, all regulated by IFN types I and II) were differentially expressed in both uveitis groups (**Figure 4C**), differing on regulation among groups (**Figure 4D**). These genes exhibited the same pattern of expression: CR1 and GNLY were downregulated, while CST7 was upregulated (**Supplementary Tables S2**, **S3**).

We conducted a functional enrichment analysis to identify specific Gene Ontology Biological Process (BP) enriched by the distinctive IRG expression signatures identified in each uveitis group (**Figures 4D, E**, **Supplementary Tables S4**, **S5**). In the uveitis only group, there was a robust enrichment of BPs related to the inflammatory response, primarily involving genes regulated by IFN types I and II. Among them are BPs enriched by upregulated DEGs involved in the production of cytokines (e.g., IL8, IL1b, and IFN gamma, beta, and alpha) and TLR signaling pathways (**Figure 4E**). In contrast, the systemic disease-associated uveitis group showed BPs enriched by upregulated DEGs associated with macrophage activation, leukocyte-mediated activation, IL-1β production, dendritic cell migration and chemotaxis, and cell killing, while several enriched BPs were associated with T cell activation and differentiation, along with those involving the production of specific cytokines, such as IFN-gamma and IL-2 (**Figure 4F**). We then further explored pathway associations of the intersecting IRGs using pathDIP, and Pathway Association Prediction (PAP) highlighted both known and novel associations related to IFN pathways, underscoring the involvement of inflammatory cytokines production and antiviral mechanism with uveitis (**Supplementary Table S6**). These predicted associations provide deeper insights into the molecular mechanisms underlying uveitis either alone or associated with systemic diseases.

### 3.4 Distinct interferome signatures in different forms of uveitis

**Figure 5:**
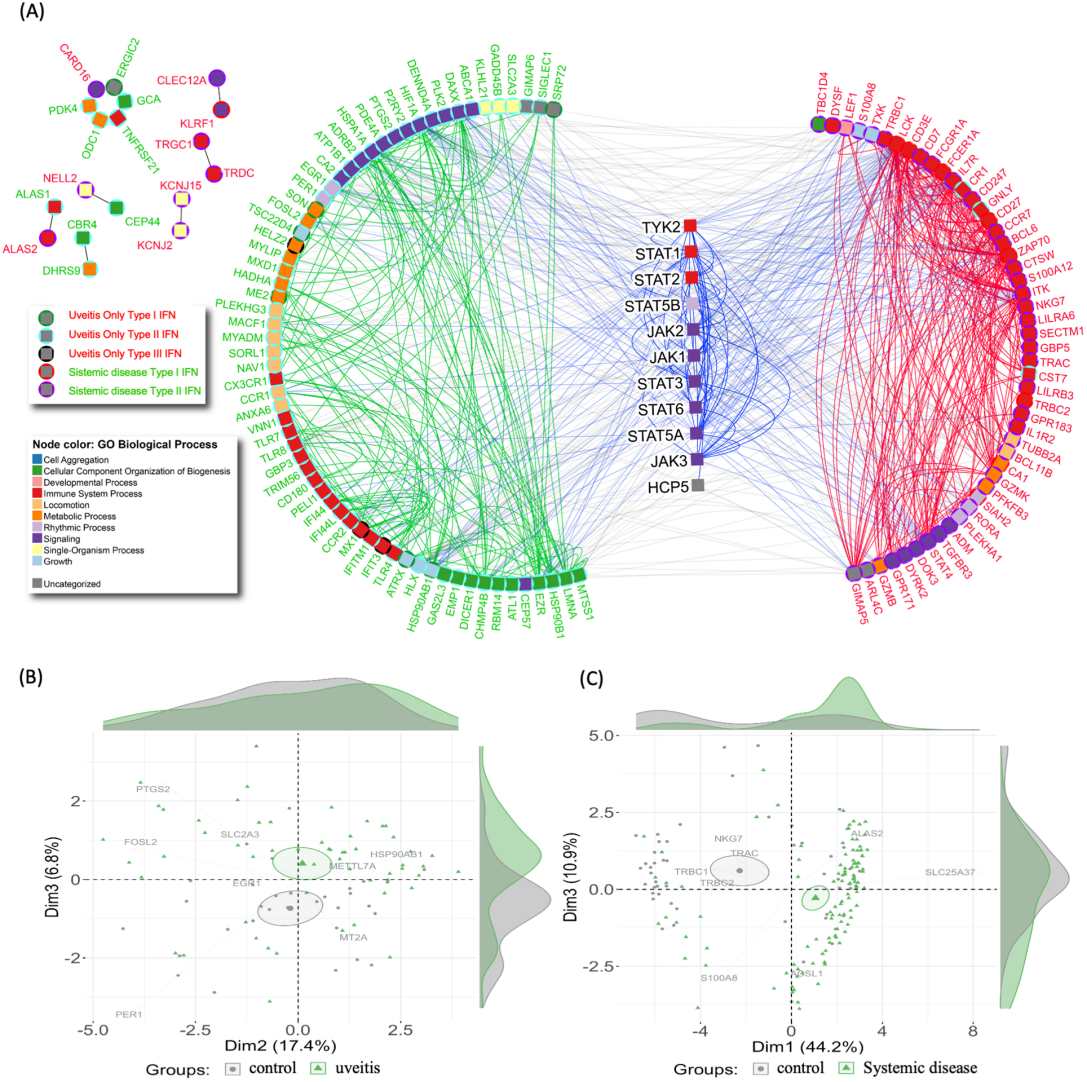
Interferome network and patient stratification in uveitis. **(A)** Protein-protein interaction network of Interferon-associated genes. Node colors indicate Gene Ontology Biological Processes. Gene names are colored to represent uveitis only (red) and systemic disease-associated uveitis group (green). Centered symbols depict genes within the JAK/STAT signaling pathway. The upper left subnetwork displays interactions among genes not associated with the main hub. **(B, C)** Principal component analysis (PCA) of Interferon-associated genes from **(B)** uveitis group and **(C)** systemic disease-associated uveitis group, distinguishing healthy controls (grey) from patients (green).

Our findings indicate systemic involvement and a complex interferome signature specific to each group of uveitis. Consistent with current research, including ongoing clinical trials and documented case reports exploring therapeutic strategies targeting JAK/STAT signaling as a novel treatment approach for both groups of uveitis (**Supplementary Table S9**), we found a specific but interconnected network of IRGs modulated by IFN types I, II, and III in both uveitis groups (**Figure 5A**).

Principal component analysis (PCA) was utilized to investigate these findings further. The PCA results indicated that the IFN signature effectively stratifies uveitis patients from healthy control individuals within each uveitis group (**Figures 5B, C**). This stratification demonstrates the significant variations in IFN signatures between uveitis patients and healthy controls, highlighting the underlying immunological differences. The stratification from healthy controls was more pronounced in the systemic disease-associated uveitis group (**Figure 5C**). This suggests systemic uveitis may involve more distinct or severe immune responses than the uveitis only. The ability of PCA to highlight these differences underscores the importance of the IFN signature in distinguishing between disease states, and, additionally, may identify potential targets for therapeutic intervention.

### 3.5 Specific interferome genes are key classifiers for the different forms of uveitis

**Figure 6:**
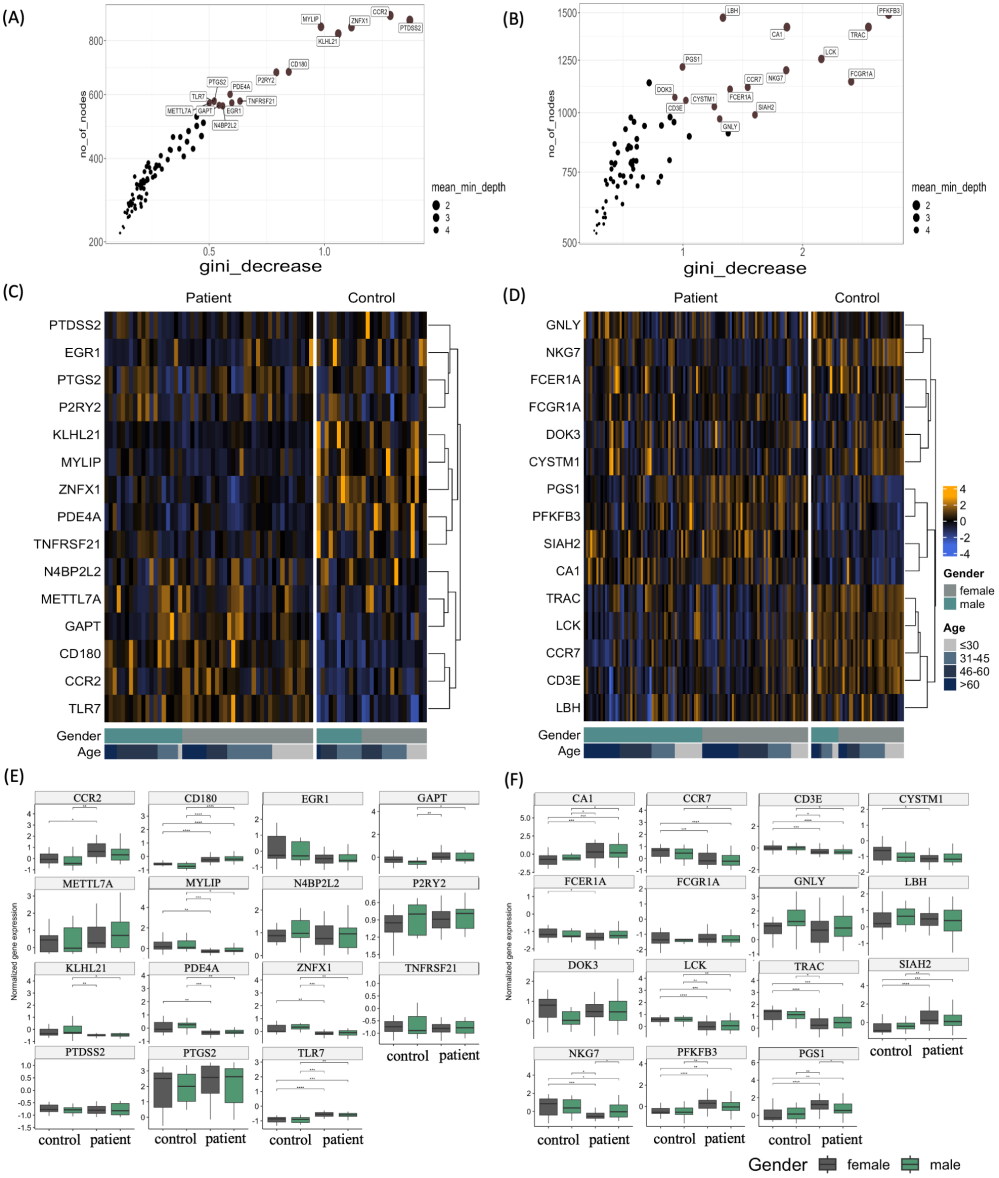
Top interferon-regulated genes ranked by Random Forest. **(A, B)** Variable importance score plots obtained by random forest classification analysis for **(A)** uveitis group and **(B)** systemic disease-associated uveitis group. These plots display the importance score based on the Gini decrease and number of nodes for each variable (Interferon-associated genes), highlighting the top 15 variables predicting the patient group compared to the healthy control group. **(C, D)** Heatmaps depicting the expression levels of the top 15 variables predicting the patient group compared to the healthy control group by random forest for **(C)** uveitis group and **(D)** systemic disease-associated uveitis group. The yellow color scale represents up-regulated genes, while the blue color scale indicates down-regulated genes. Heatmaps are categorized by healthy control individuals and patients, age groups of >60, 46-60, 31-45, and <30 years old, and gender. **(E, F)** Box plots of differentially expressed genes (DEGs) of Uveitis patients compared to healthy controls, according to sex in uveitis only group and systemic disease-associated uveitis group. *p ≤ 0.05;**p ≤ 0.01;***p ≤ 0.001;****p ≤ 0.0001.

To further investigate the interferome’s capacity to stratify uveitis patients based on disease manifestation, we applied the machine learning algorithm Random Forest (RF) to identify gene classifiers of uveitis (**Figure 6**). This approach allowed us to rank the most critical variables within each group (**Supplementary Figures S1**–**S3**). Consistent with the PCA results, the RF analysis of uveitis patients versus healthy individuals revealed an out-of-bag (OOB) error rate of 13.33% and an area under the curve (AUC) of the ROC curves of 0.9 for the uveitis-only group. We found an OOB error rate of 16.07% and an AUC of 0.68 for the systemic disease-associated uveitis group (**Supplementary Figures S4A–D**). These findings demonstrate the high accuracy of the Random Forest analysis (**Supplementary Figures S4E–H**), suggesting that IRGs are robust classifiers of uveitis when presented as a solo clinical manifestation. The top 15 IRG classifiers are shown from least to most significant in the upper right corner of the graphs (**Figures 6A, B**). The expression patterns of these genes are shown in **Figures 6C, D**, highlighting both downregulated and upregulated genes. For instance, in the uveitis only group, the genes *KLHL21, MYLIP, ZNFX1, PDE4A, TNFRSF21*, and *N4BP2L2* were downregulated, while *METTL72, GAPT, CD180, CCR2*, and *TLR7* were upregulated when comparing uveitis patients with healthy controls (**Figure 6C**). In the systemic disease-associated group, the genes *PGS1, PFKFB3*, *SIAH2*, and *CA1* were upregulated, and *TRAC, LCK, CCR7, CD3E*, and *LBH* were downregulated when comparing patients with healthy controls (**Figure 6D**).

Moreover, in the uveitis only group, the expression of several genes was differentially regulated according to gender and age. Females showed a significant upregulation of CCR2, CD180, and TLR7 and downregulation of MYLIP, PDE4A, and ZNFX1 compared to healthy individuals of the same sex group (**Figure 6E**). Male individuals exhibited different upregulated genes, such as *CD180, GAPT*, and *TLR7*; meanwhile, a downregulation of *KLHL21, MYLIP, PDE4A*, and *ZNFX1*. A sex-dependent expression of genes was also detected among the systemic disease-associated uveitis patients, with the genes *CA1, CCR7, CD3E*, and *LCK* being differentially regulated in male and female patients compared with healthy individuals of the same sex. Genes such as *CYSTM1, FCER1A, NKG7, PFKFB3, PGS1, SIAH2*, and *TRAC* are exclusively differently expressed among female patients and healthy individuals (**Figure 6F**). Furthermore, there was a significant correlation between patients’ age-specific regulation of genes *MYLIP* and *PDE4A* in the uveitis only group and *CCR7, LBH*, and *LCK* in the systemic disease-associated uveitis group (**Supplementary Figure S5**).

### 3.6 Uveitis risk factors and predictive markers

To further investigate the 15 genes identified as the strongest classifiers of both uveitis groups, we performed Linear Discriminant Analysis (LDA). The clinical classification of healthy controls versus uveitis patients was the dependent variable. At the same time, the gene expression levels of the top RF genes served as the independent variables to study their specificity and sensitivity. The cut-off threshold was established based on specificity, sensitivity, and accuracy parameters, aiming for a correct group classification rate of preferably 70% (**Supplementary Tables S10**, **S11**). Additionally, the Risk Ratio (RR) was calculated from the obtained cut-off values, providing deeper insights into the relationship between the dependent variable (group classification) and the independent variable (gene expression levels) to predict the likelihood of developing uveitis.

**Figure 7:**
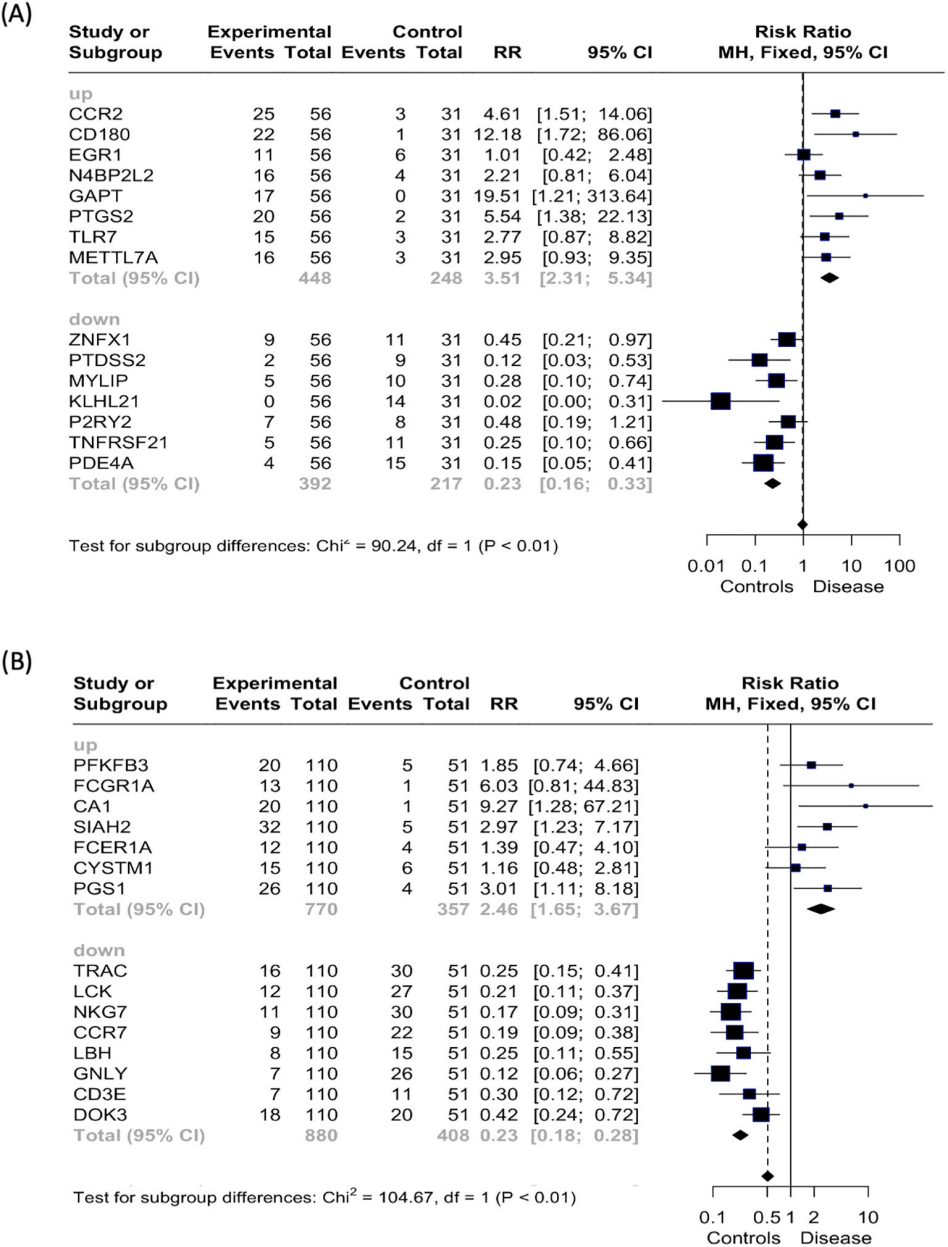
Risk ratio (RR) analysis. Forest plot displaying the risk ratio (RR, represented by squares) and 95% confidence interval (CI, indicated by horizontal lines) for each of the top 15 variables predicting the patient group compared to the healthy control group by random forest analysis for the **(A)** uveitis only group and **(B)** systemic disease-associated uveitis group. The vertical dotted line signifies a risk ratio representing “even odds”.

The genes that showed a significantly increased risk ratio for uveitis in the uveitis only group, and were upregulated in patients, included *CCR2, CD180, GAPT*, and *PTGS2* (**Figure 7A**). Downregulated genes with a significantly increased risk ratio for uveitis in the same group were *PTDSS2, MYLIP, KLHL21, TNFRSF21*, and *PDE4A*. In the systemic disease-associated uveitis group, the statistically significant upregulated genes associated with an increased risk ratio for uveitis were *CA1, SIAH2*, and *PGS1*, while all downregulated predictive genes were above the cut-off (**Figure 7B**).

## 4 Discussion

The development of autoimmune diseases is influenced by a diverse range of factors. However, an additional layer of complexity arises in immune-privileged sites such as the eye. Here, antigens are sequestered behind blood-tissue barriers, limiting their exposure to the immune system and impeding the induction of peripheral tolerance (54). This unique immunological environment predisposes the eye to immune dysregulation, contributing to the distinct pathophysiology of uveitis

Immune-mediated non-infectious uveitis is a clinically diverse group of ocular conditions that share immune characteristics similar to those seen in systemic autoimmune and autoinflammatory disorders (55). Recent advancements in the detection of proinflammatory cytokines have substantially deepened our understanding of their role in the pathogenesis of uveitis and identified cytokines as IFN and IRG signatures that are among the most essential features of the immunological response which often results in inflammation and autoimmune diseases (56, 57). However, characteristics of IRGs in non-infectious uveitis patients have not been evaluated. Our current study emphasizes the predominance of IRGs among metaDEGs generated from diverse datasets, characterizing the interferome signature as an evident hallmark of the immune response in non-infectious uveitis. Moreover, this work demonstrates a different IRG signature according to the specific clinical manifestation of uveitis.

Modular co‐expression analysis combined with co-expression enrichment and network analyses identified a strong association of cytokine signaling (e.g. regulation of interleukins and interferon) in both groups of uveitis evaluated. Specific cytokines may be linked to a particular inflammatory pathway, disease activity, or response to a specific therapeutic approach (58–60). Studies using intraocular samples have been of great value in the investigation of the role of cytokines in the development of uveitis, revealing in patients’ aqueous humor a predominance of pro-inflammatory and vascular mediators such as IL-6, IL-8, G‐CSF, TNF-α, and VEGF, (59, 61, 62). The same study observed an elevated concentration of IL-17, IL-10 and IL-21 in patients’ serum, indicating the involvement of Th1/IL-21–Th17 pathways, as well as increased levels of IFN-γ-inducing cytokine IL-12, and IFN-γ-inducible CXC chemokine IP-10. In addition to the involvement of an extensive array of cytokines, a number of studies support the hypothesis that there is a difference in immunological responses when uveitis is part of a broader systemic disease, such as VKH disease, ocular sarcoidosis, Behcet's disease, or when established as the only clinical manifestation (17, 63, 64).

Our meta-analysis of datasets referring to uveitis alone and systemic disease-associated uveitis highlights the deep involvement of cytokines and chemokines signaling pathways, either as receptors for specific molecules or as part of the broader interferon-mediated immune response. It reinforces the distinction of differentially expressed genes according to the group of uveitis. In the systemic disease-associated uveitis group, in addition to identifying signaling pathways such as the chemokine receptor CCR7, which is implicated in T cell homing (65), we detected metaDEGs associated not only with T cell responses, but also with B cell responses. The latter includes receptors for the immunoglobulin Fc region involving all immunoglobulin isotypes, and members of the leukocyte immunoglobulin-like receptor (LILR) family. In accordance, immunologic assessment of syndrome-associated uveitis forms, such as VKH disease, revealed the activation of CD4+ T lymphocyte-mediated cellular immunity (66) and the infiltration of B lymphocytes and plasma cells into the vitreous, suggesting the participation of humoral immunity (67). While B cell responses may contribute to systemic disease associated uveitis, in the uveitis only presentation, cytokines like IFN, growth factors, and interleukins could play dual roles in the pathogenesis, serving as both pro-inflammatory and anti-inflammatory agents (68). We were able to identify a number of IFN-associated metaDEGs in both groups, including several genes encoding proteins directly involved in the IFN-induced response (e.g., IFIT3, IFI44, IFI44L, IFITM1, MX1, and DHRS9) (69). These genes have previously been implicated in mediating and regulating inflammatory processes in different manifestations of uveitis (70, 71).

The investigation of IRGs in both forms of uveitis allowed us to dissect the importance of singular interferome dynamics in each group. In this context, we identified more than 80% of IRGs among the metaDEGs of each group, with only 3 IRGs overlapping in both groups. The type of IRGs was not a significant factor since there was almost no difference between the groups, except for type III IFN – associated genes identified in the uveitis-only group, but not in the systemic disease-associated uveitis group.

Among the most statistically significant IRGs, the uveitis-only group exhibited a higher proportion of upregulated genes, whereas the systemic disease-associated uveitis group showed a predominance of downregulated genes. Only three genes overlapped between the two groups: *CST7* (Leukocystatin), *GNLY* (Granulysin - Antimicrobial peptide), and *CR1* (Complement receptor type 1). Notably, *GNLY* and *CR1* displayed inverse regulation patterns, being upregulated in the uveitis-only group but downregulated in the systemic disease-associated uveitis group. The *CR1* gene was previously connected to uveitis associated with sarcoidosis due to a gene polymorphism thought to affect disease susceptibility, implicating a genetic predisposition (72). Several studies have demonstrated that the complement system not only contributes to systemic disease-associated uveitis, but also plays a critical role in the pathogenesis of experimental autoimmune anterior uveitis, endotoxin-induced uveitis (73, 74), as well as in acute anterior uveitis (75). However, there has not been a well-established association between uveitis and *CST7* or *GNLY*.

To further explore the specificity of each interferome signature for each uveitis group, we performed stratification and classification analyses (PCA and random forest). Some of the genes that were identified as important classifiers of the uveitis only group, such as *P2RY2* and *CCR2*, are known to be relevant in the pathogenesis of uveitis. Cells with high expression of CCR2 have previously been demonstrated in the eye fluid of uveitis patients, while the absence of these gene products attenuates eye lesions through decreased cytokine secretion or reduced uveitis antigen-specific T-cell activation (29, 76, 77). Another target of immunotherapy herein predicted as uveitis classifier is the gene *PDE4A*, coding for the intracellular non-receptor enzyme, phosphodiesterase 4A, known to modulate inflammation, including T cell-induced inflammation. Inhibitors of phosphodiesterase 4A have been investigated as potential therapeutic agents for the treatment of autoimmune uveitis and Behçet disease, one of the uveitis-associated systemic diseases (78, 79).

The imperative role of T cells in systemic disease-associated uveitis is reinforced by the characterization of disease-related genes identified in this study. The gene *CD3E*, which we found to be downregulated in systemic disease-associated uveitis, encodes the CD3 epsilon subunit, which forms, together with other CD3 subunits, the T-cell receptor (TCR)-CD3 complex (80). Upon TCR engagement, this complex becomes phosphorylated, activating downstream signaling pathways and thus initiating the disease process. Unlike what was observed for the systemic disease-associated uveitis group, this pathway blockage has been shown to reduce symptom severity (81) on experimental autoimmune uveitis. As participants in the T cell response cascade, *TRAC* and *LCK* genes, which we have identified as disease predictors, are also involved in T cell signaling through the recognition of processed small peptides bound to MHC molecules, a process previously associated with Behçet disease by transcriptome evaluation (82).

The importance of the chemokine signaling pathway in the etiology of this group of uveitis is highlighted by the differentially expressed gene, *CCR7*, encoding a receptor expressed in lymphoid tissues associated with T and B lymphocyte activation, control of memory T cell migration and the activation and polarization of T cells in the pathogenesis of chronic inflammation (82). Although the *CCR7* gene is downregulated in the systemic-associated uveitis group, mouse models of autoimmune uveitis demonstrate an increase of Natural Killer (NK) cells expressing CCR7 in inflamed eyes, associated with elevated expression of NKG2D, CD69, and IFN‐γ (83), which may highlight the differences in the inflammatory processes between the two groups.

One of the key pathways through which cytokines, IFNs, growth factors, and signaling molecules mediate their effects is the phosphorylation of JAK-STAT proteins (84). Evidence from both human non-infectious uveitis and experimental uveitis models indicates a crucial role of the JAK-STAT signaling pathway in disease pathogenesis (85–88). Several case reports suggest that JAK-STAT inhibitors, particularly JAK1, JAK2, and JAK3, may serve as a promising therapeutic strategy, especially in refractory cases that do not respond adequately to conventional or biological treatments. Patients receiving this therapy have demonstrated good tolerance and symptom remission. One proposed mechanism underlying this therapeutic effect involves the SIAH2 gene, identified in our study as a potential disease predictor for systemic disease-associated uveiti, which encodes an E3 ubiquitin-protein ligase that modulates the JAK-STAT pathway (89), by regulating the stability and activity of proteins involved in this pathway. Another potential mechanism is a direct association with T-cell activity, as blocking key components of T-cell priming, specifically STAT1 and STAT3, has been shown to attenuate or suppress effector T-cell activation and migration, thereby reducing ocular inflammation and tissue damage (35, 90, 91). Additionally, genetic factors may contribute to this regulation, as genetic variants in STAT3, STAT4, and JAK1 have been associated with an increased risk of ocular involvement in patients with Behçet’s disease (85, 92, 93).

To classify uveitis as non-infectious requires the exclusion of any active infection. The role of microbes in the etiology of autoimmunity, and the role of the microbiome in triggering autoimmune or autoinflammatory reactions have been intensely investigated. Although we did not directly evaluate the role of the microbiome, it is likely that endogenous microbes participate in disease development. In this context, two of the genes we identified as predictors in the uveitis only group, *TLR7* and the TLR4 homologue, *CD180*, were reported previously to undergo activation following the binding of specific antigens originating from viruses or bacteria, resulting in the initiation of the immunological cascade (94). Metagenomic studies have shown differentially abundant gut microbiota composition as well as specifically altered metabolites in VKH, Behcet disease, and acute anterior uveitis (95–97), with a microbial or viral infection preceding the onset of diseases (98, 99). However, the causal relationship requires future studies since active infections could not be diagnosed at the time of sample collection.

An important aspect highlighted in this study is the sex-dependent regulation when comparing gene expression by individuals of the same sex from both the patient and control groups. In the systemic disease-associated uveitis group, we observed a notable disparity in DEGs between patients and healthy individuals of the same sex. Specifically, 73% of the RF-predicted genes were differentially regulated in females, while only 27% were in males, with a higher proportion of downregulated genes in female patients than males. This pattern contrasts with findings in Behçet’s disease, where most interferon-responsive genes (IRGs) in neutrophils were downregulated, predominantly in male patients (100). Conversely, in the uveitis-only group, the distribution of differentially regulated genes was more balanced, with 40% in females and 47% in males, showing an approximately equal proportion of upregulated and downregulated genes between sexes. Epidemiological studies have shown that sex plays a role in the presentation and progression of certain autoimmune diseases, such as spondyloarthritis, one of the systemic disease-associated uveitis, with women having higher tendency to a more severe disease course (101). It has been postulated that genetic and hormonal mechanisms, particularly estrogen due to its known role in modulating immune responses, may influence the immune system’s response, often enhancing the production of antibodies and inflammatory cytokines, potentially contributing to this disparity (102, 103). Data obtained from non-infectious uveitis further emphasize sex differences, indicating that sex can influence not only the likelihood of developing different types of uveitis but also the response to treatments with women having less favorable prognosis (7).

We acknowledge that our study relies on bioinformatics analyses to characterize the interferome signature in uveitis and systemic disease-associated uveitis. The integrative systems biology approach employed in our study enabled the identification of key DEGs and their associated pathways, providing valuable insights into the molecular mechanisms underlying these conditions. One limitation is the reliance on publicly available transcriptomic datasets, which may introduce variability due to differences in sample collection, experimental protocols, and data processing methods across studies. Additionally, while bioinformatics approaches allow for large-scale data integration and statistical robustness, they cannot fully capture the complexity of immune responses at the protein level or in specific cellular subtypes that may be underrepresented in transcriptomic datasets. The findings presented here, particularly those related to differentially expressed IRGs, should be interpreted in the context of these constraints. Furthermore, future experimental validation using functional assays and single-cell transcriptomic approaches will be essential to confirm the observed immune signatures and provide a more granular understanding of uveitis pathophysiology.

Another limitation of the present study is that unlike what is observed in systemic disease-associated uveitis, the datasets available for the uveitis-only group were obtained from studies focusing on specific cell types, such as monocytes and dendritic cells. Due to this limitation, our findings predominantly reflect the activation of the innate immune system in the uveitis-only group. However, we cannot rule out the possibility of dual activation of both the innate and adaptive immune systems in cases where uveitis is the sole manifestation. This suggests that the immunological response in uveitis-only patients may differ from that observed in systemic disease-associated uveitis, highlighting potential distinctions in the underlying immune mechanisms between these conditions.

Additionally, our blood transcriptome analysis revealed a differential expression profile enriched IFN-associated genes in uveitis groups. Whether these expression patterns parallel those observed in ocular tissues remains highly relevant, yet transcriptomic data from the eye are currently limited. For instance, the study by Errera et al. (61) investigated the protein profile of aqueous humor in patients with idiopathic uveitis, demonstrating a significant increase in IFN-γ and TNF-α, among other pro-inflammatory cytokines. These findings reinforce the concept of inflammatory pathway activation within the ocular microenvironment, particularly highlighting the involvement of interferon signaling, which aligns with our blood transcriptomic data. Although this study did not directly examine IRGs in ocular tissues, the presence of IFN-γ and other inflammatory mediators in the aqueous humor implies that key immune mechanisms are shared between the blood and the eye. This is particularly relevant given that interferon signaling plays a pivotal role in shaping both innate and adaptive immune responses in uveitis. Therefore, the IFN-associated transcriptional signature identified in our blood transcriptome analysis may reflect not only systemic immune dysregulation but also intraocular immune activity, suggesting that peripheral blood gene expression patterns could serve as a surrogate marker for inflammatory responses in target ocular tissues. Further investigations incorporating transcriptomic profiling of ocular samples are needed to validate this systemic-ocular connection and explore its potential diagnostic and therapeutic implications.

Our findings contribute to unraveling the complex pathogenesis of systemic disease-associated uveitis and uveitis as an exclusive manifestation, highlighting key molecular pathways involved in disease progression. Specifically, identifying IFN-signaling pathways as potentially central players in uveitis pathophysiology underscores their role in shaping immune responses across different disease subtypes. Moreover, the interferome signatures described in this study provide valuable insights into the immune landscape of non-infectious uveitis, paving the way for refined diagnostic approaches and personalized therapeutic strategies tailored to distinct clinical presentations. These findings reinforce the need for further investigations to validate blood-based transcriptional signatures as potential biomarkers for disease classification, prognosis, and treatment response in patients with uveitis.

## Data Availability

The original contributions presented in the study are included in the article/**Supplementary Material**. Further inquiries can be directed to the corresponding authors.
