# Supplementary material for: Integrative immunology identified interferome signatures in uveitis and systemic disease-associated uveitis
Source: Front Immunol. 2025 Apr 9;16:1509805. doi: 10.3389/fimmu.2025.1509805 (PMC12014655; doi:10.3389/fimmu.2025.1509805)
Supplement: Supplementary file 2 [file DataSheet2.pdf]

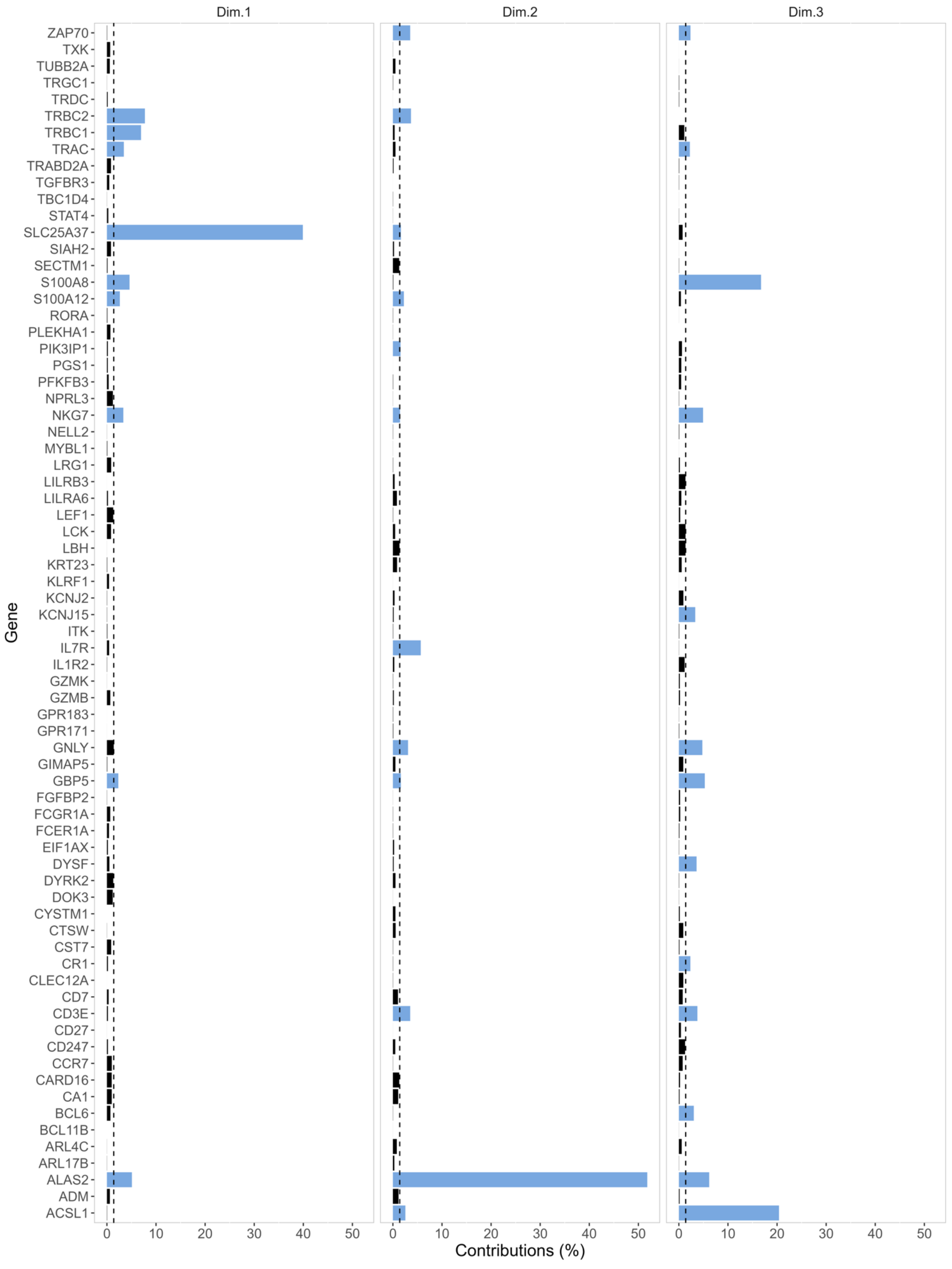

Figure S2. Barplot with the contribution percentages of each variable to each dimension in the PCA of systemic disease-associated uveitis group. A black dashed line is plotted on the 5% mark, and blue bars indicate a contribution higher than 5%.
