# Supplementary material for: Integrative immunology identified interferome signatures in uveitis and systemic disease-associated uveitis
Source: Front Immunol. 2025 Apr 9;16:1509805. doi: 10.3389/fimmu.2025.1509805 (PMC12014655; doi:10.3389/fimmu.2025.1509805)
Supplement: Supplementary file 3 [file DataSheet3.pdf]

(A) (B)

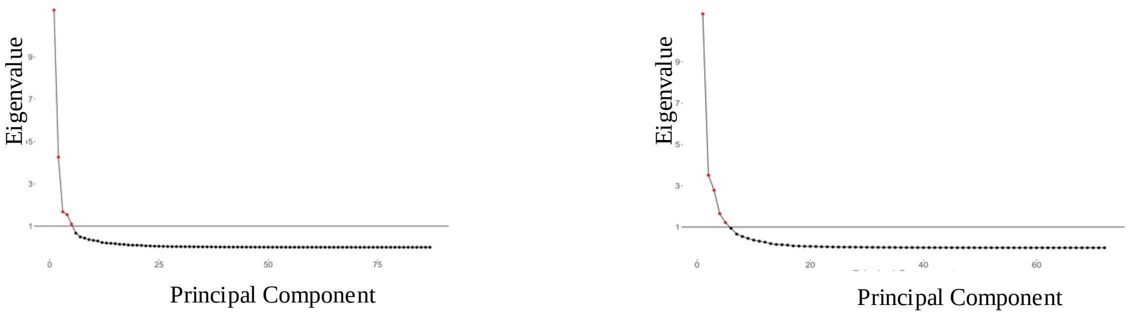

Figure S3. Principal Component Analysis. Eigenvalue of Principal component analysis (PCA) of Interferon-associated genes from (A) uveitis group and (B) Systemic disease-associated uveitis group.
