# Supplementary material for: Integrative immunology identified interferome signatures in uveitis and systemic disease-associated uveitis
Source: Front Immunol. 2025 Apr 9;16:1509805. doi: 10.3389/fimmu.2025.1509805 (PMC12014655; doi:10.3389/fimmu.2025.1509805)
Supplement: Supplementary file 4 [file DataSheet4.pdf]

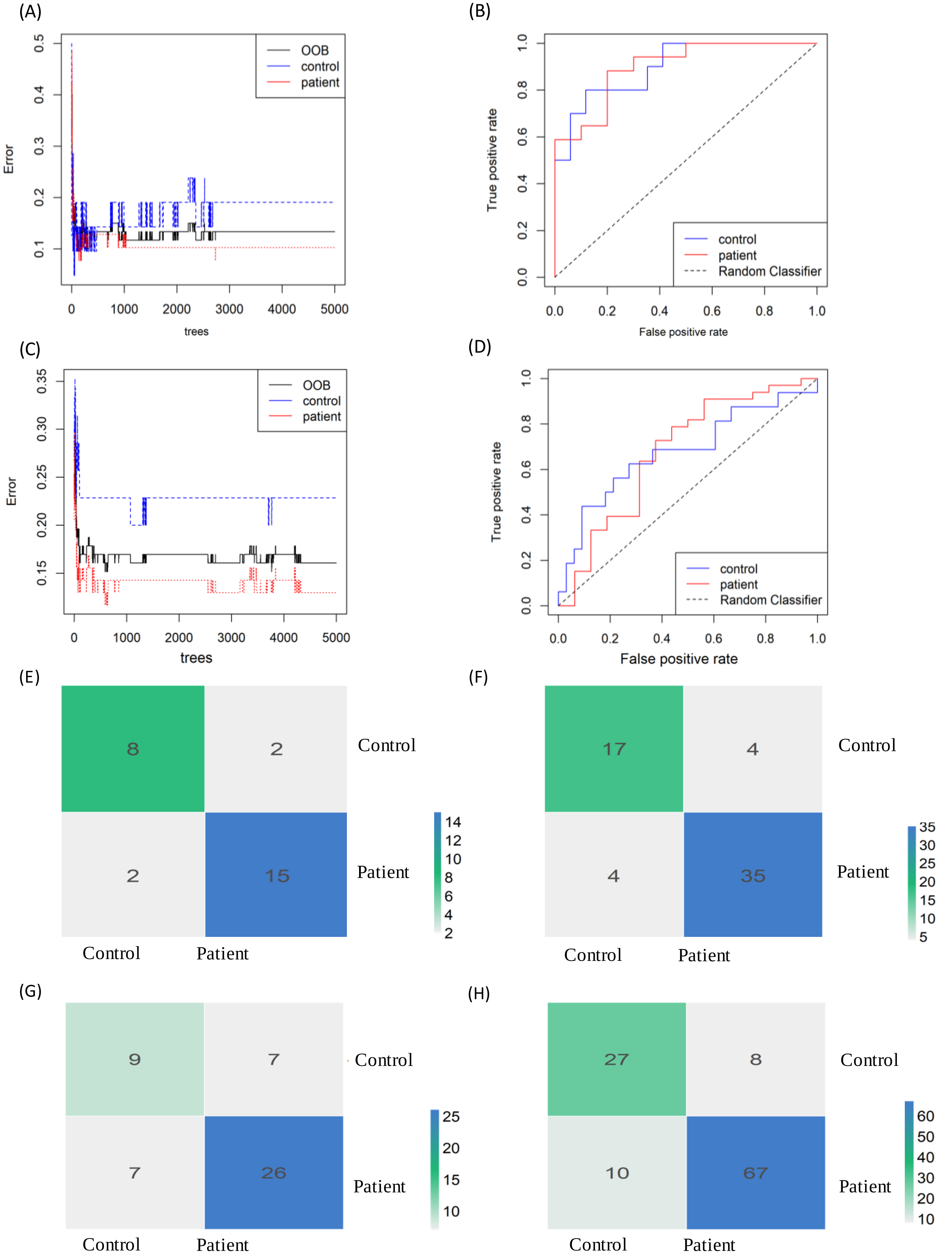

Figure S4. Control parameters of Random Forest analysis. Stable curve indicating the number of trees and out-of-bag (OOB – black line) error rate of the random forest analysis for the uveitis and systemic disease-associated uveitis groups (A and C, respectively). Receiver operating characteristic (ROC) curves with an area under the curve (AUC) exhibiting the relationship between true and false positive classification rates for uveitis group and systemic disease-associated uveitis group (B and D, respectively). The blue line indicates healthy controls, and the red line indicates patients. Heatmap of the confusion matrix of training (E-F) and testing (G-H) in the uveitis group (E-G) and systemic disease-associated uveitis group (F-H). Numbers represent the amount of occurrences that happened when training the random forest model in predicted (row) vs actual classification (column), therefore the blueish diagonal identifies the hits, while other cells are mismatches.
