# Supplementary material for: Integrative immunology identified interferome signatures in uveitis and systemic disease-associated uveitis
Source: Front Immunol. 2025 Apr 9;16:1509805. doi: 10.3389/fimmu.2025.1509805 (PMC12014655; doi:10.3389/fimmu.2025.1509805)
Supplement: Supplementary file 5 [file DataSheet5.pdf]

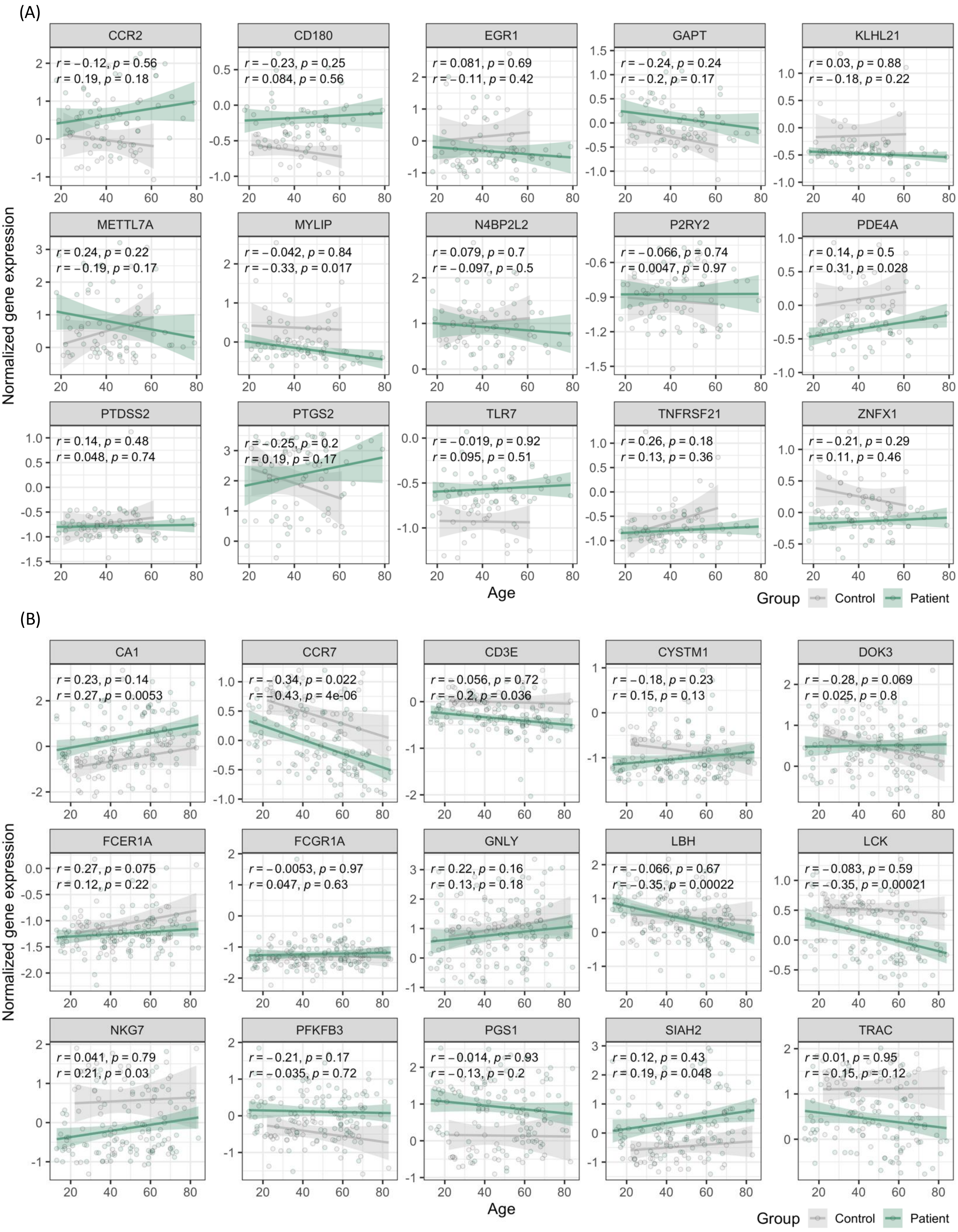

Figure S5. Correlation between top interferon-regulated gene expression ranked by RF across group and age. Scatter plots with correlation of normalized gene expression with age in (A) uveitis group and (B) systemic disease-associated uveitis group, with fitted linear regression lines representing control (gray) and patient (green). Correlation coefficient (r) and significance level (p-value) for each correlation on control and patients group are shown within each graph.
